# Low Hydration Heat with High Strength in LHPC Composite Binders Governed by Hydration Efficiency and Matrix Densification

**DOI:** 10.3390/ma19091824

**Published:** 2026-04-29

**Authors:** Pengyu Cai, Yanfeng Zuo, Zhongcheng Ma, Hongxia Wang, Junhua Guo, Chunyong Gao, Yun Liu, Minglin Jia, Chengzhong Gui, Hongchuan Chen, Chen Wang, Yuetong Yi

**Affiliations:** 1 School of Earthquake Engineering and Building Safety, University of Emergency Management, No. 465 Xueyuan Street, Yanjiao High-Tech Zone, Sanhe 065201, China; 17806247128@163.com (M.J.); 13115287@bjtu.edu.cn (C.G.); chenhongchuanchc@163.com (H.C.); 13081032098@163.com (C.W.); sikamio@163.com (Y.Y.); 2 China Building Materials Academy Co., Ltd., No. 1 Guanzhuang Dongli, Chaoyang District, Beijing 100024, China; mazhongcheng.com@163.com (Z.M.); wxx022@126.com (H.W.); guojunhua870108@163.com (J.G.); gaobao1124@163.com (C.G.); cbmliuyun@163.com (Y.L.); 3 Key Laboratory of Building Collapse Mechanism and Disaster Prevention, China Earthquake Administration, Institute of Disaster Prevention, Sanhe 065201, China; 4 Hebei Provincial Technology Innovation Center for Multi-Hazard Resilience and Emergency Response of Engineering Structures, Institute of Disaster Prevention, Sanhe 065201, China; 5 Langfang Key Laboratory of Engineering Structures Anti-Collapse, Institute of Disaster Prevention, Sanhe 065201, China

**Keywords:** low-heat Portland cement (LHPC), supplementary cementitious materials (SCMs), heat of hydration, isothermal calorimetry, hydration kinetics, Krstulović–Dabić model, bound water, microstructural densification

## Abstract

**Highlights:**

- A low-heat–high-strength LHPC-based binder was achieved via multi-SCM synergy.
- Hydration efficiency, rather than total heat, governs strength development.
- Matrix densification was confirmed by XRD, TGA, and SEM analyses.

**Abstract:**

Achieving low hydration heat without sacrificing strength is essential for early-age temperature-crack control in concrete. This study designed a low-heat Portland cement (LHPC)–fly ash (FA)–ground-granulated blast-furnace slag (GGBS)–silica fume (SF) binder system with LHPC fixed at 80 wt.% and total supplementary cementitious materials (SCMs) fixed at 20 wt.%. Compressive strength at 3, 7, and 28 d, 7 d isothermal calorimetry combined with Krstulović–Dabić (K–D) modeling, X-ray diffraction (XRD), thermogravimetric analysis (TGA), and scanning electron microscopy (SEM) were used to identify a low-heat/high-strength pathway. The mixture containing 20 wt.% FA (F20) reduced the 7 d cumulative heat to 194.060 J·g^−1^ but lowered the 28 d compressive strength to 44.2 MPa. Replacing FA with GGBS under the same replacement level restored the strength baseline, and the mixture containing 20 wt.% GGBS (G20) reached 56.7 MPa. Introducing SF created an optimum compositional window, and the mixture containing 10 wt.% FA, 3 wt.% GGBS, and 7 wt.% SF (F10G3S7) achieved the highest 28 d strength of 58.2 MPa. Notably, the mixture containing 10 wt.% FA, 9 wt.% GGBS, and 1 wt.% SF (F10G9S1) combined relatively low 7 d heat (203.545 J·g^−1^) with high 28 d strength (54.2 MPa). K–D fitting showed that FA lowered the heat potential (Qmax = 217.98 J·g^−1^) relative to LHPC (236.19 J·g^−1^), whereas GGBS/SF blends increased Qmax to 268.77–271.55 J·g^−1^, indicating composition-dependent hydration efficiency. TGA revealed higher bound water per unit LHPC at 28 d (21.46–22.97%) than in LHPC alone (17.17%), and bound water correlated more strongly with compressive strength (R^2^ = 0.75–0.78) than calcium hydroxide (CH) content (R^2^ = 0.66–0.67). SEM confirmed a more continuous gel-rich matrix in F10G9S1, suggesting that the low-heat/high-strength route is governed by efficient heat-to-hydrate conversion and microstructural densification rather than heat output alone. These findings provide both mechanistic insight and practical guidance for proportioning low-heat, high-strength binders for concrete applications requiring early-age temperature-crack control.

## 1. Introduction

Early-age thermal cracking remains a key durability risk for concrete structures, especially under massive casting or restrained boundary conditions [1,2,3]. The fundamental driving force is the temperature rise and gradient induced by cement hydration heat, which can generate tensile stresses during cooling and shrinkage, thereby accelerating crack initiation and propagation [4,5]. Consequently, developing cementitious systems with reduced hydration heat while maintaining sufficient mechanical performance is essential for temperature-crack control and low-carbon construction [6,7].

Low-heat Portland cement (LHPC) is an effective approach to suppress heat release by tailoring clinker reactivity; however, the accompanying penalty in early-age strength and the uncertainty in long-term performance often limit its wider engineering adoption [7,8]. Mineral admixtures, such as fly ash (FA), ground granulated blast-furnace slag (GGBS), and silica fume (SF), are frequently incorporated to regulate both heat evolution and strength development [8,9,10]. FA typically exhibits dilution and retardation effects, decreasing early heat output but potentially lowering the strength baseline [11]. GGBS provides latent hydraulic reactivity and can contribute to pronounced later-age strength growth [12]. SF, characterized by ultrafine particle size and high amorphous SiO_2_ content, may enhance pore refinement and interfacial densification through filler and pozzolanic effects [13]. Although multi-component blending is widely considered promising due to potential synergy, the coupled “heat release–hydration progression–microstructural densification–strength development” relationships in LHPC-based binders remain insufficiently clarified, particularly within constrained design domains with fixed total replacement [14].

In practice, mixture optimization toward a “low-heat yet high-strength” pathway is still largely experience driven, and reproducible guidelines are scarce [15,16]. Existing studies often focus on binary blends or vary replacement levels simultaneously, making it difficult to isolate compositional effects in multi-component systems and to identify efficient hydration-to-strength conversion routes [17]. More importantly, systematic linkage among calorimetric kinetics, hydration products, and microstructural evidence is still limited, which restrains mechanism-informed mix design [18].

Therefore, this study constructs an LHPC–FA–GGBS–SF multi-component binder system by fixing LHPC at 80% and the total mineral admixture content at 20%. A series of mixtures with different FA/GGBS/SF proportions are designed and evaluated through an integrated macro-to-micro framework: compressive and flexural strengths at 3, 7, and 28 days; isothermal calorimetry and Krstulović–Dabić (K–D) kinetic modeling to quantify hydration progression; and XRD, TGA, and SEM analyses to reveal hydration products and densification pathways. The objective is to elucidate the governing mechanism of the “heat potential–hydration rate–effective hydrate formation–microstructural refinement–strength gain” chain and to provide practical guidance for designing LHPC-based binders with a balanced low-heat/high-strength performance.

However, previous studies on low-heat cementitious systems have mainly focused on binary or loosely controlled blended binders, in which replacement level, binder composition, and reactivity often vary simultaneously, making cross-comparison difficult and obscuring the intrinsic roles of individual supplementary cementitious materials (SCMs) in governing heat release and strength development. Although it is generally recognized that fly ash (FA) tends to suppress early heat evolution, ground granulated blast-furnace slag (GGBS) can enhance later-age reactivity, and silica fume (SF) may improve matrix densification; a systematic understanding of how these SCMs interact in constrained LHPC-based multi-component systems is still lacking. In particular, the mechanistic link between calorimetric response, hydration-product development, and microstructural refinement has not been sufficiently established, which limits the development of reliable mixture-design principles for achieving low hydration heat without sacrificing strength. In this context, the present study provides new insight by examining a fixed-design-domain LHPC–FA–GGBS–SF system and clarifying how heat potential, hydration progression, effective hydrate formation, and matrix densification collectively govern strength gain. Therefore, the primary objective of this work is to identify a feasible low-heat/high-strength compositional pathway and to establish a mechanism-informed basis for proportioning LHPC-based binders for early-age temperature-crack control.

## 2. Materials and Methods

### 2.1. Materials and Sample Preparation

#### 2.1.1. Materials

Low-heat Portland cement (denoted as LHPC), fly ash (FA), ground granulated blast-furnace slag (GGBS), and silica fume (SF) were employed as the constituent binders in this study. The chemical compositions of LHPC, FA, GGBS, and SF were determined by X-ray fluorescence spectroscopy (XRF) (PANalytical Axios, PANalytical B.V., Almelo, The Netherlands). The contents of major oxides are summarized in Table 1, while the corresponding elemental compositions are reported in Table 2. As shown in Table 1, CaO and SiO_2_ are the predominant oxides in LHPC; FA is mainly composed of SiO_2_ and Al_2_O_3_; GGBS contains relatively high proportions of both CaO and SiO_2_; and SF is characterized by its exceptionally high SiO_2_ content.

**Figure 1:**
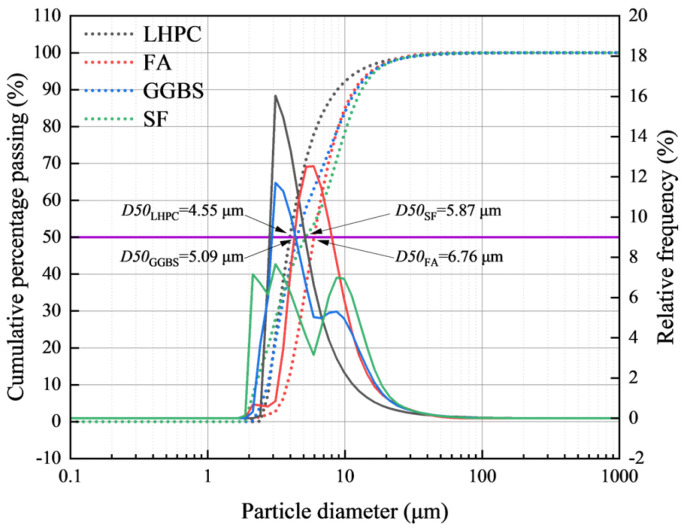
Particle size distribution of LHPC, FA, GGBS and SF *. * D50 represents the median particle diameter, meaning that 50% of the particles are smaller than this size and 50% are larger. The solid lines represent the full particle size distribution curves of the four raw materials plotted against the right y-axis (relative frequency).

The particle size distributions of the four raw materials were measured using a laser particle size analyzer (Mastersizer 3000, Malvern Instruments Ltd., Malvern, Worcestershire, UK). The characteristic particle size parameters together with the full distribution curves are presented in Figure 1.

**Figure 2:**
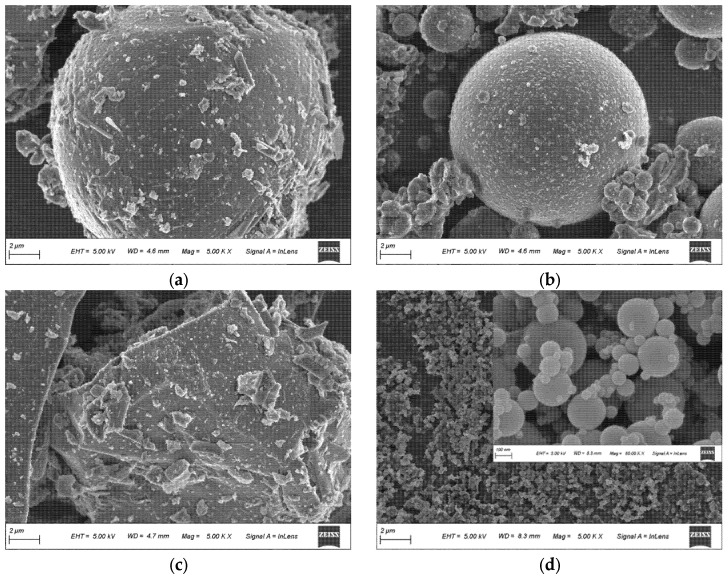
SEM micrographs of raw cementitious materials: (**a**) LHPC; (**b**) FA; (**c**) GGBS; (**d**) SF.

To further characterize the particle morphology of the raw materials, scanning electron microscopy (SEM) (FEI Quanta 250 FEG) (Thermo Fisher Scientific, Hillsboro, OR, USA) was conducted for LHPC, FA, GGBS, and SF, and the representative micrographs are shown in Figure 2. The SEM images were primarily used to identify particle shape, surface texture, and the attachment of fine particles, thereby providing a reference baseline for subsequent comparisons with hydration products and microstructural evolution.

#### 2.1.2. Sample Preparation

All binder systems investigated in this study were designed within a fixed compositional domain. The reference mixture was pure low-heat Portland cement (LHPC), while all blended systems were prepared by fixing LHPC at 80 wt.% and the total supplementary cementitious material (SCM) content at 20 wt.%. The SCMs used in this study included fly ash (FA), ground granulated blast-furnace slag (GGBS), and silica fume (SF). According to the number and proportion of SCMs incorporated, the investigated binders can be categorized into binary, ternary, and quaternary systems. The mixture nomenclature was defined based on the mass fractions of FA, GGBS, and SF in the binder. For example, F20 denotes a binary system containing 20 wt.% FA; F15G5 and F10G10 denote ternary systems containing FA and GGBS at mass ratios of 15:5 and 10:10, respectively; and F10G9S1 denotes a quaternary system containing 10 wt.% FA, 9 wt.% GGBS, and 1 wt.% SF. The detailed binder compositions of all investigated mixtures are summarized in Table 3.

The total SCM content was fixed at 20 wt.% to establish a controlled and practically relevant design domain in which the effects of FA, GGBS, and SF proportions could be compared without interference from changes in the overall replacement level. This replacement level was selected to ensure a measurable reduction in hydration heat while avoiding excessive dilution of the LHPC matrix, thereby maintaining a reasonable strength baseline for subsequent comparison. Under this fixed total replacement, the influence of progressively substituting FA with GGBS and SF could be examined more clearly, allowing the compositional contribution of each SCM to hydration heat evolution and strength development to be identified.

For the tests on mechanical performance and drying shrinkage, mortar specimens were prepared by mixing binder, water, and ISO standard sand at prescribed mass proportions, with the water-to-binder (w/b) ratio fixed at 0.50. Specifically, each mortar mixture consisted of 450 ± 2 g of binder, 225 ± 1 g of water, and 1350 ± 5 g of ISO standard sand. Cement paste specimens for X-ray diffraction (XRD), thermogravimetric analysis (TGA), and scanning electron microscopy (SEM) were also prepared at a w/b ratio of 0.50 without adding standard sand. In contrast, paste specimens for hydration-heat measurements were prepared separately at a lower w/b ratio of 0.40, also without standard sand.

Mortar prisms with dimensions of 40 mm × 40 mm × 160 mm were cast for compressive and flexural strength tests in accordance with GB/T 17671–2021 [19] and cured in water at 20 ± 1 °C until the designated ages. For drying shrinkage tests, mortar specimens with dimensions of 25 mm × 25 mm × 280 mm were prepared and cured at 20 ± 3 °C and a relative humidity of 50 ± 4%. At the specified curing ages, hardened cement pastes were crushed, and hydration was terminated using absolute ethanol. The collected fragments were then dried at 40 °C for 3 days. For XRD and TGA analyses, the dried samples were ground to pass an 80 μm sieve (i.e., particle size < 80 μm), whereas SEM observations were conducted directly on the dried specimens without further grinding.

### 2.2. Characterization Methods

#### 2.2.1. Mechanical Properties

The flexural and compressive strengths of mortar specimens were determined in accordance with GB/T 17671–2021 [19]. For each mixture and curing age, three mortar prisms (40 mm × 40 mm × 160 mm) were tested for flexural strength, and the six half-prisms obtained after flexural failure were subsequently used for compressive strength testing. The reported values represent the mean results of three flexural specimens and six compressive specimens.

#### 2.2.2. Hydration Heat

The hydration heat of cement pastes was measured using an isothermal calorimeter (TAM Air) (TA Instruments, New Castle, DE, USA). For each binder system, the heat-flow rate and cumulative heat release were continuously recorded for 7 d. Based on the calorimetric results, the early-age hydration behavior was evaluated, including the magnitude of the main heat-release peak and its time of occurrence.

#### 2.2.3. XRD

The phase assemblages of hydration products in paste samples were analyzed using an X-ray diffractometer (Rigaku D/MAX series) (Rigaku Corporation, Tokyo, Japan) with Cu Kα radiation. The diffraction patterns were collected over a 2θ range of 5–90° at a scanning rate of 6°/min. XRD analysis was performed to identify the presence and relative evolution of major crystalline phases formed during hydration, such as calcium hydroxide (Ca(OH)_2_, CH) and unreacted components.

#### 2.2.4. TGA

Thermogravimetric analysis of hydrated paste samples was carried out using a Q500 thermogravimetric analyzer (TA Instruments, New Castle, DE, USA) under an argon atmosphere, with a sample mass of approximately 10 ± 1 mg. The temperature was increased from room temperature to 1000 °C at a heating rate of 10 K/min. Based on the mass-loss features observed in the thermogravimetric (TG) and derivative thermogravimetric (DTG) curves, the decomposition intervals and corresponding weight-loss contributions of hydration products were identified. The calcium hydroxide content and bound-water content were first quantified as *w*_CH_ and *w*_H_, respectively. To enable comparison among mixtures with different LHPC contents, the corresponding LHPC-normalized indices, *I*_CH_ and *I*_H_, were further calculated using Equations (1)–(4):

(1) $$w_{CH}(\%)=WL_{CH}(\%)\cdot \frac{MW_{CH}}{MW_{H_{2}O}}+WL_{CC}(\%)\cdot (\frac{MW_{CH}}{MW_{CO_{2}}}),$$

(2) $$I_{CH}(\%)=w_{CH}(\%)\cdot \frac{100}{w_{LHPC}(\%)},$$

(3) $$w_{H}(\%)=\frac{W_{40}-W_{550}}{W_{550}},$$

(4) $$I_{H}=w_{H}\frac{100}{w_{LHPC}(\%)},$$

where *WL*_CH_ and *WL*_CC_ denote the weight losses associated with calcium hydroxide (CH) dehydroxylation and calcium carbonate (CaCO_3_) decarbonation, respectively; *MW*_H2O_, *MW*_CO2_, and *MW*_CH_ are the molecular weights of H_2_O, CO_2_, and CH, respectively; *W*_40_ and *W*_550_ represent the sample masses at 40 °C and 550 °C, respectively; and *w*_LHPC_(%) is the mass percentage of LHPC in the binder, equal to 100% for the LHPC mixture and 80% for all blended mixtures. Here, *I*_CH_ and *I*_H_ represent the calcium hydroxide content and bound-water content normalized to the LHPC fraction in the binder, respectively [20,21,22,23].

Because hydration was terminated by solvent exchange using absolute ethanol and the fragments were subsequently dried at 40 °C, a small amount of residual ethanol or ethanol–water mixture could remain trapped in fine pores at early ages. Therefore, any sharp low-temperature mass-loss event below 100 °C was interpreted with caution and was not directly assigned to hydrate decomposition alone.

#### 2.2.5. Microstructure

Hydration of the paste specimens was terminated by solvent exchange with anhydrous ethanol, followed by drying, and the resulting microstructure was examined using a field-emission scanning electron microscope (FEI Quanta 250 FEG) (Thermo Fisher Scientific, Hillsboro, OR, USA). SEM observations were conducted to qualitatively assess key morphological features, including hydration-gel characteristics, portlandite crystal features, residual supplementary cementitious material particles and their reaction extent, as well as pore/microcrack defects and local densification in the interfacial transition zone (ITZ).

Unless otherwise specified, data processing, linear regression fitting, and figure plotting were performed using OriginPro 2022 (OriginLab Corporation, Northampton, MA, USA).

## 3. Results

### 3.1. Mechanical Properties

**Figure 3:**
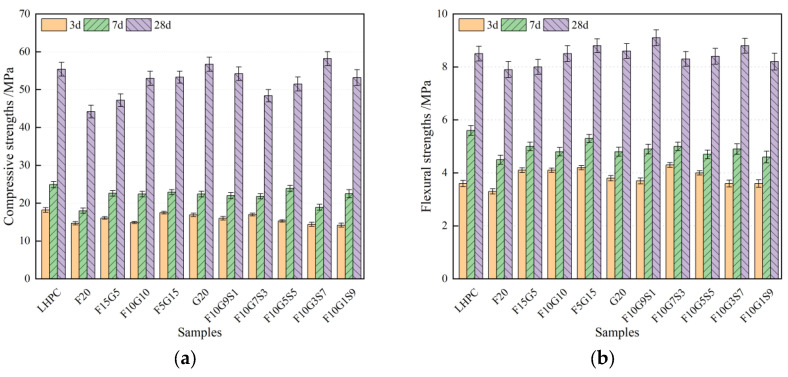
Mechanical properties of different samples: (**a**) compressive strength; (**b**) flexural strength.

Figure 3 shows that replacing part of cement with fly ash reduces both compressive and flexural strengths, with early-age strength being particularly affected. For the FA-only mixture (F20), the strength reduction remains evident even at 28 d. In contrast, replacing FA with GGBS improves later-age strength, and the 28 d compressive strength is largely restored in slag-containing mixtures. The incorporation of silica fume further modifies the strength response and reveals an effective dosage window, whereas excessive SF addition does not continuously improve strength. Overall, these results indicate that FA tends to weaken early-age mechanical performance, while GGBS and an appropriate amount of SF can compensate for this loss and enhance later-age strength development [24,25,26,27]. Nonetheless, previous studies have also shown that the influence of FA on strength is highly dependent on mixture design, activation condition, and curing regime; under optimized conditions, FA-containing systems may still achieve acceptable early-age strength, and flexural performance may improve at later ages [28]. The corresponding statistical summary of the flexural and compressive strength results, including mean values, standard deviations (SD), and coefficients of variation (COV), is provided in Appendix A (Table A1 and Table A2).

### 3.2. Hydration Heat

**Figure 4:**
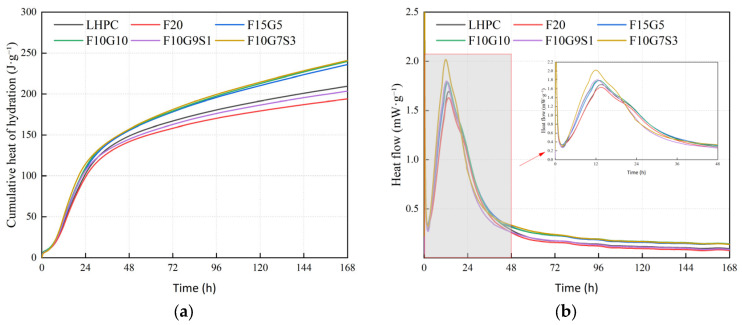
Hydration heat evolution of LHPC-based binders with different binder compositions: (**a**) cumulative heat; (**b**) heat flow.

As shown in Figure 4a, all binders exhibit a rapid increase in cumulative heat release at early ages, followed by a gradual slowdown, while the magnitude of heat release is strongly governed by SCM type and reactivity. Replacing cement with fly ash tends to suppress early heat evolution and marginally prolong the induction-to-acceleration transition, leading to lower cumulative heat at both 3 d and 7 d; in this study, F20 remains below LHPC at 3 d and 7 d. This early-age heat reduction is consistent with the commonly reported dilution and retardation effects of fly ash in isothermal calorimetry [29].

For slag- and silica-fume-containing blends, the early heat signature is more sensitive to particle-driven nucleation/filler effects and to the availability of sulfates/alkalis that activate latent hydraulic or pozzolanic reactions. Accordingly, slag incorporation can shift the main hydration peak to earlier times and increase the main peak intensity relative to fly-ash-rich blends, while the cumulative heat at 7 d may increase when the slag is sufficiently reactive. In the present results, the blended mixtures show earlier main-peak times than LHPC/F20 and a wider 7 d cumulative-heat range. It should be noted that slag is often used to reduce temperature rise in mass concrete; however, several studies have cautioned that modern, more reactive or sulfate-bearing slags may exhibit comparable or even higher heat release than other SCMs under certain calorimetric conditions, which aligns with the elevated heat output observed in some slag-rich mixtures here [30]. The key isothermal calorimetry parameters, including the time to the main peak, main peak heat flow, and cumulative heat at 3 d and 7 d, are summarized in Table 4.

### 3.3. Hydration Products

#### 3.3.1. XRD

**Figure 5:**
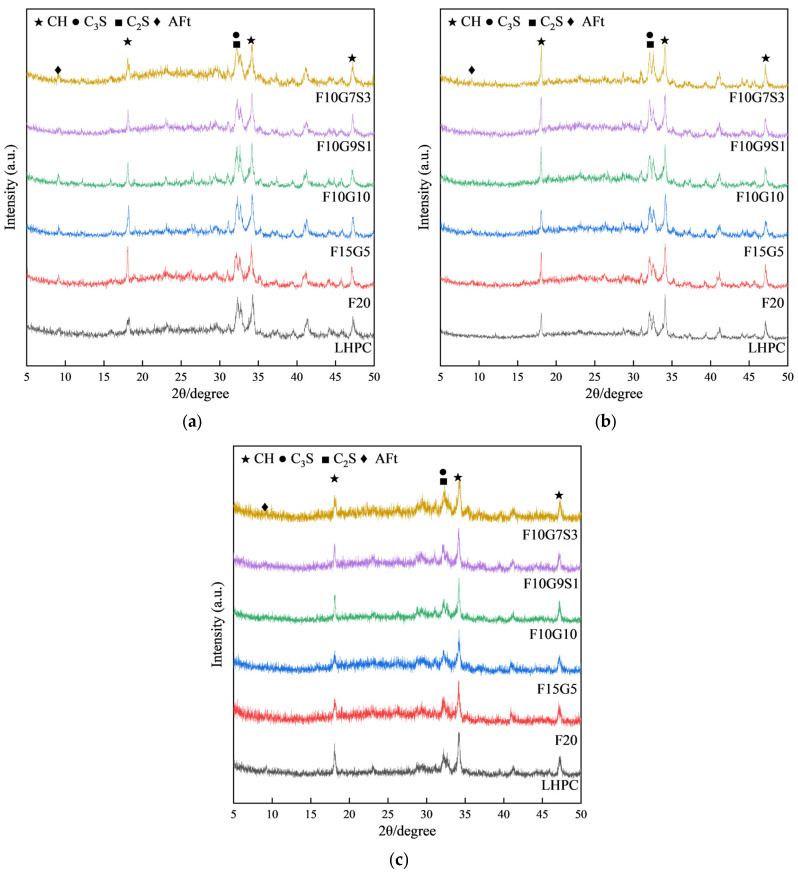
XRD patterns of LHPC and blended binders at different curing ages: (**a**) 3 d; (**b**) 7 d; (**c**) 28 d.

Figure 5a–c present the XRD patterns of LHPC and the blended binders at 3, 7, and 28 days. In all systems, diffraction peaks attributable to portlandite and ettringite can be identified, while distinct reflections of unhydrated clinker phases remain evident at early ages, indicating incomplete hydration at 3 days. With increasing curing age, the overall phase evolution reflects continuous hydration and product accumulation, which is consistent with the general understanding that the incorporation of supplementary cementitious materials primarily modifies the kinetics and efficiency of hydrate development rather than changing the fundamental hydrate assemblage [31].

From the perspective of material effects, the early-age response can be rationalized by the relatively slow pozzolanic reactivity of fly ash, which tends to delay the development of strength-contributing hydrates at early ages, whereas later-age benefits arise from secondary gel formation that densifies the matrix. In contrast, silica fume, owing to its ultrafine and highly amorphous nature, can react more rapidly with portlandite and also provide a strong filler effect, thereby accelerating microstructural densification within an effective dosage window; correspondingly, XRD may exhibit a reduced portlandite peak intensity in aluminosilicate-bearing systems. For GGBS, its contribution is often associated with latent hydraulic and pozzolanic-type reactions, and its fineness is a key factor governing the extent and rate of reaction with Ca(OH)_2_, which provides a mechanistic basis for the observed phase-development differences among blended systems across curing ages [32].

#### 3.3.2. TGA

**Figure 6:**
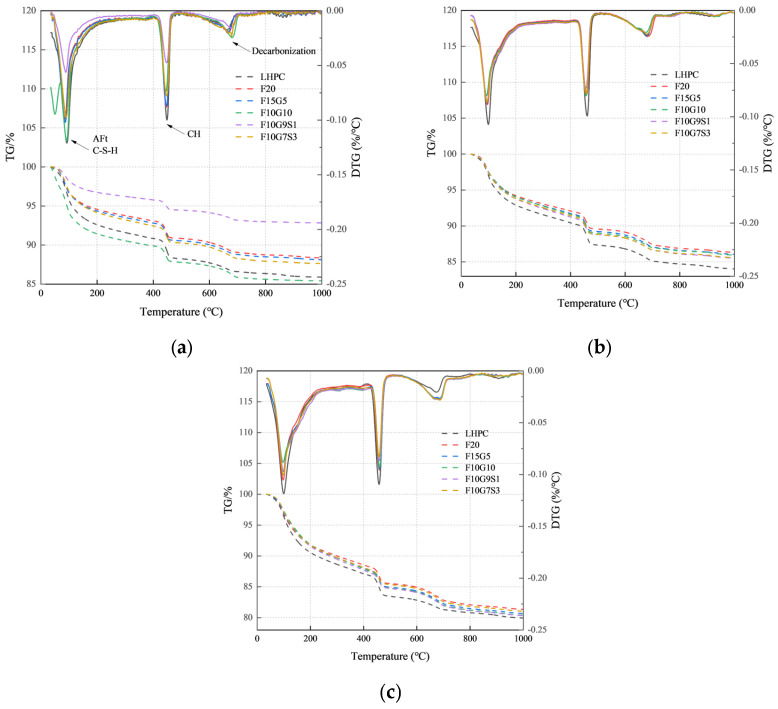
TG and DTG results in different samples: (**a**) 3 d; (**b**) 7 d; (**c**) 28 d *. * The solid lines represent the full DTG curves of the samples plotted against the right y-axis (DTG).

Figure 6 illustrates the thermogravimetric (TG) and derivative thermogravimetric (DTG) curves of LHPC and blended systems, where mass loss is attributed to AFt and C-(A)-S-H dehydration at low temperatures, CH dehydroxylation around 430–470 °C, and decarbonation of carbonate phases, mainly attributed to CaCO_3_, at higher temperatures [33,34]. From these data, CH and bound-water loss (H) were quantified and normalized (*I*_CH_ and *I*_H_), as shown in Table 5. Most blended systems show equal or higher *I*_CH_ at 7 and 28 days compared to LHPC, suggesting promoted LHPC hydration per unit LHPC (nucleation/filler effects), rather than per unit binder through filler or nucleation effects. In contrast, F10G9S1 presents notably lower *I*_CH_ at 3 days, suggesting suppressed CH formation or faster consumption due to silica fume. Increasing the SF content further reduces *I*_CH_ at 28 days, pointing to intensified late-age pozzolanic activity. *I*_H_ rises significantly from 7 to 28 days in all blends, especially in F20 and FA–GGBS systems, confirming active pozzolanic and latent hydraulic reactions in later stages [35]. F10G9S1 also shows a sharp increase in *I*_H_ between 3 and 7 days, reflecting delayed but accelerated hydration. Overall, the TGA results demonstrate that multi-component blending improves later-age hydrate formation and modulates CH development, supporting performance gains at 28 days.

The low-temperature peak below 100 °C, especially for the 3 d F10G10 sample, is likely affected by residual ethanol or ethanol–water volatilization; therefore, the corresponding early-age *I*_H_ values should be interpreted with caution.

### 3.4. Microstructure

**Figure 7:**
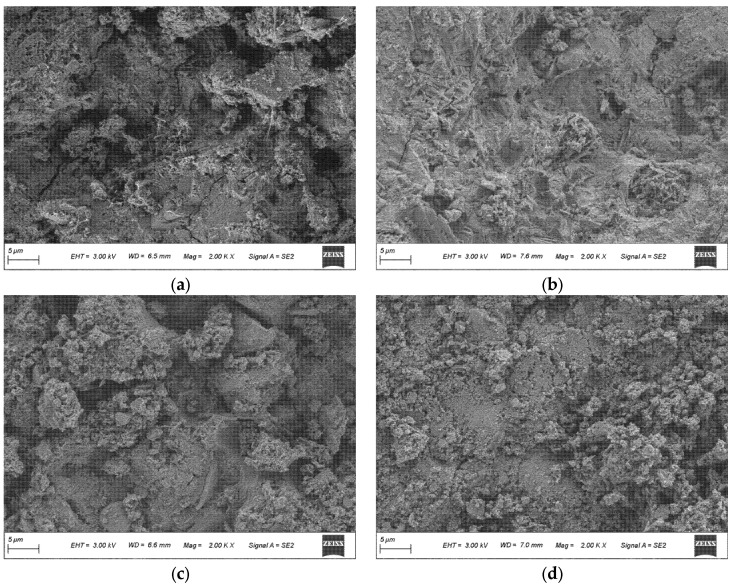

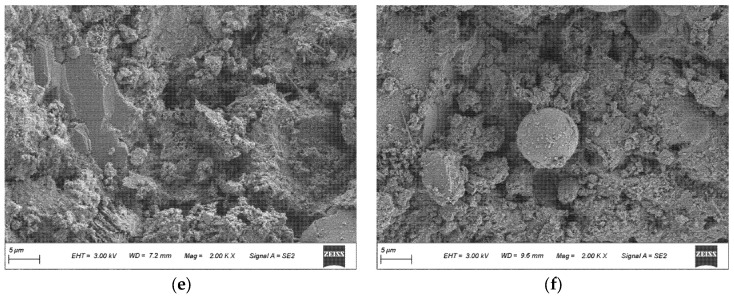
SEM micrographs of six representative blends at 28 d: (**a**) LHPC; (**b**) F20; (**c**) G20; (**d**) F10G10; (**e**) F10G9S1; (**f**) F10G7S3.

**Figure 8:**
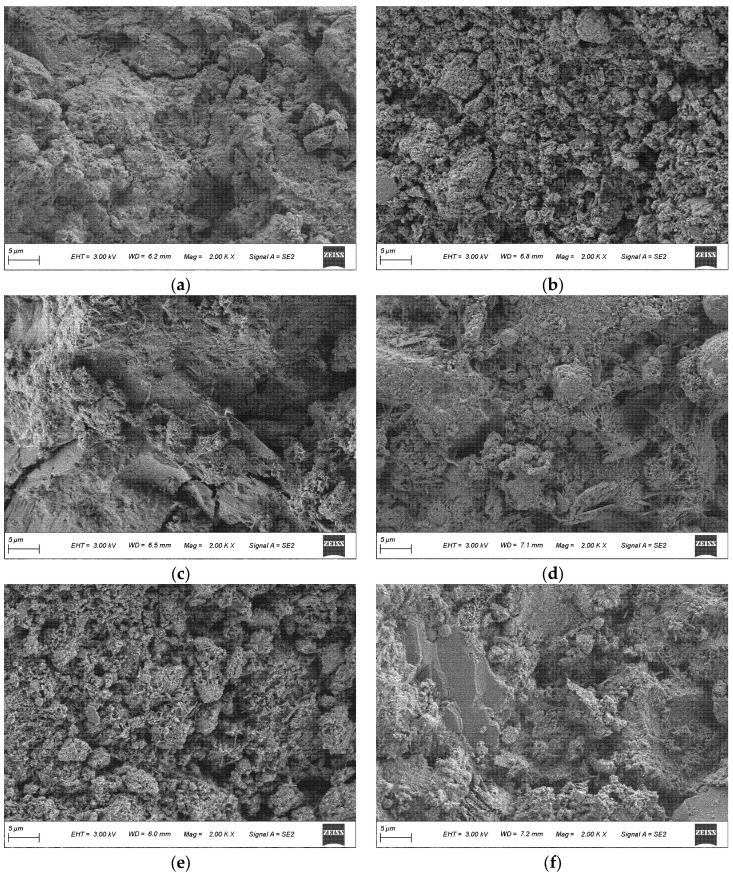
SEM micrographs of LHPC and F10G9S1 at different curing ages: (**a**) LHPC-3 d; (**b**) LHPC-7 d; (**c**) LHPC-28 d; (**d**) F10G9S1-3 d; (**e**) F10G9S1-7 d; and (**f**) F10G9S1-28 d.

The SEM observations in Figure 7 and Figure 8 are interpreted mainly in terms of matrix compactness, residual particle features, pore/void distribution, and the continuity of hydration products.

Figure 7 presents SEM micrographs of six representative binders at 28 d, enabling a direct comparison of matrix compactness and the distribution of unreacted particles and voids at the same curing age. Overall, LHPC exhibits a more integrated and compact matrix. In contrast, the FA-containing system F20 tends to show a more open framework with a higher presence of unreacted particulate features, which agrees with the widely reported strength reduction associated with FA dilution and its limited early-age contribution to solid skeleton formation [36]. Introducing slag, as in G20 and FA–slag blends, is expected to densify the microstructure through additional hydrate formation. This is because slag can consume CH and promote the formation of additional C–A–S–H, thereby filling pores and refining the interfacial and matrix microstructure [37]. In addition, incorporating highly reactive fine SCMs such as silica fume, as in F10G9S1 and F10G7S3, can further enhance microstructural refinement by consuming CH and providing nucleation and filler effects. These mechanisms accelerate hydrate formation and contribute to a more compact microstructure [38].

Figure 8 compares the age-dependent microstructural evolution of LHPC and F10G9S1 from 3 d to 28 d. For LHPC, the matrix progressively densifies with curing age, reflecting continuous hydration and gradual pore filling by hydration products [36]. Compared with LHPC, F10G9S1 tends to exhibit a more heterogeneous and relatively looser microstructure at early ages. This is largely because a considerable portion of fly ash can remain unreacted by 28 d in high-volume FA-related systems, acting predominantly as an inert filler in the early stage and delaying densification. With prolonged curing, F10G9S1 becomes more compact as slag-driven hydraulic and pozzolanic reactions consume CH and generate additional C–A–S–H to fill pores. Meanwhile, silica fume can further promote densification through CH consumption as well as nucleation and filler effects [39]. This age-dependent evolution provides a mechanistic explanation for the commonly observed pattern of early-age weakness followed by accelerated later-age gain in FA–slag–silica fume blended systems [38].

## 4. Discussion

### 4.1. Hydration Kinetics

**Figure 9:**
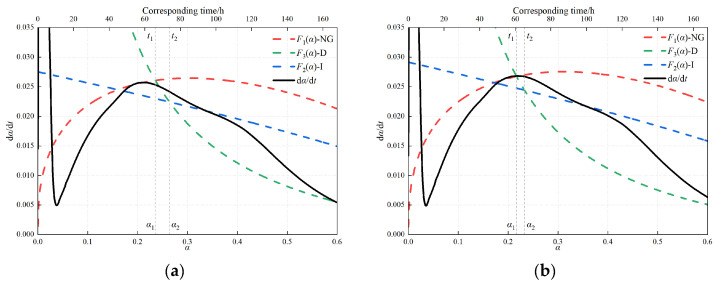

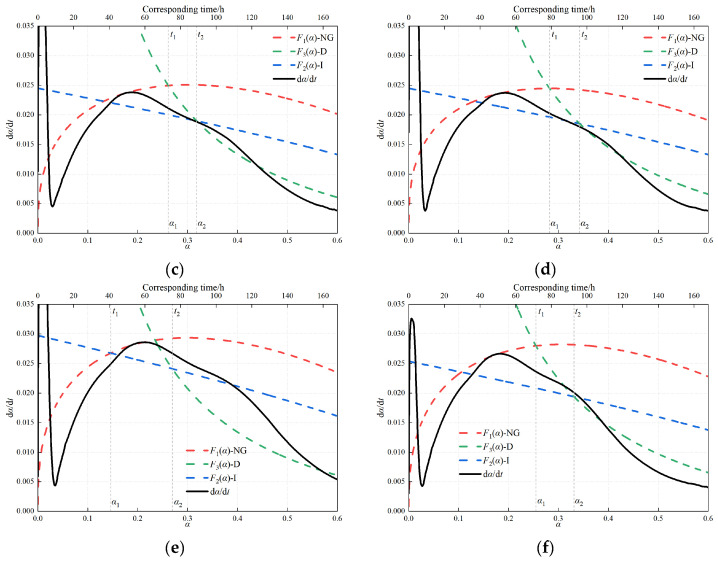
Krstulović–Dabić model-based decomposition of hydration rate curves into nucleation and crystal growth (NG), interfacial reaction (I), and diffusion (D) stages for representative LHPC-based binders: (**a**) LHPC; (**b**) F20; (**c**) F15G5; (**d**) F10G10; (**e**) F10G9S1; (**f**) F10G7S3 *. * The upper x-axis represents the approximate corresponding time associated with the degree of hydration α and is included only as an auxiliary reference for stage interpretation, not as a second independent variable for fitting.

This section discusses the hydration kinetics of the LHPC composite system using the Krstulović–Dabić (K–D) model. According to the K–D model, the hydration process of cement-based materials can be divided into three stages: nucleation and crystal growth (NG), interfacial reaction (I), and diffusion (D) [40]. Figure 9 presents the K–D model-based decomposition of the hydration-rate curves for representative LHPC-based binders under different combinations of fly ash (FA), ground granulated blast-furnace slag (GGBS), and silica fume (SF). In Figure 9, the ordinate d*α*/d*t* represents the hydration rate, the lower abscissa *α* denotes the degree of hydration, and the upper horizontal axis gives the corresponding time in hours. The black solid line represents the overall hydration-rate curve, while the dashed curves correspond to the fitted contributions of the nucleation and crystal growth stage (F_1_(*α*), NG), interfacial reaction stage (F_2_(*α*), I), and diffusion stage (F_3_(*α*), D), respectively. The characteristic points *α*_1_ and *α*_2_ indicate the transition boundaries between the NG and I stages and between the I and D stages, respectively, whereas *t*_1_ and *t*_2_ are the corresponding transition times.

The kinetic parameters obtained from K–D model fitting are summarized in Table 6. Here, *Q*_∞_ (i.e., *Q*_max_) represents the ultimate cumulative heat potential; *t*_50_ is the time required to reach 50% of *Q*_max_; *n* is the kinetic exponent associated with the nucleation-and-growth stage; and *K*_1_, *K*_2_, and *K*_3_ are the apparent rate constants for the nucleation-and-growth (NG), interfacial reaction (I), and diffusion (D) stages, respectively. For each mixture, the values reported in Table 6 are fitted kinetic parameters obtained from the average calorimetric results of three independent tests. For the K–D analysis, *t*_0_ was taken as the end of the induction period. The fitting intervals of the NG, I, and D stages were chosen from representative post-induction regions of the calorimetric curve, corresponding to the acceleration peak, the post-peak transition region, and the late low-rate tail, respectively.

Table 6 shows that the final cumulative heat potential *Q*_∞_ for each system is ordered as follows: F10G7S3 ≈ F10G10 ≈ F15G5 > LHPC > F10G9S1 > F20. Compared with LHPC, F20 shows a 7.7% decrease in *Q*_∞_, indicating that the sole use of FA effectively reduces the final heat release of the system. By contrast, F15G5, F10G10, and F10G7S3 exhibit increases in *Q*_∞_ of approximately 13.8–15.0% relative to LHPC, indicating that different combinations of GGBS and silica fume significantly alter the heat-release potential within the investigated composition range.

Meanwhile, significant differences in *t*_50_ are observed: compared with LHPC, F15G5 and F10G10 prolong *t*_50_ by 5.60 h and 5.15 h, respectively, whereas F10G9S1 and F20 shorten *t*_50_ by 2.37 h and 2.13 h, respectively. This indicates that the influence of blended systems on hydration is reflected not only in the total heat-release potential but also in the rate of hydration advancement. Regarding the stage rate constants, the *K*_1_ values follow the order F10G9S1 > F10G7S3 > F20 > LHPC > F15G5 > F10G10. Compared with LHPC, the *K*_1_ values of F15G5 and F10G10 decrease by 0.00177 and 0.00213, respectively, indicating stronger suppression of the NG-stage reaction rate. In contrast, F10G9S1 exhibits the highest *K*_1_ and the shortest *t*_50_, suggesting a more pronounced early-age acceleration process. The differences in *K*_2_ among the samples are relatively limited, whereas *K*_3_ shows larger fluctuations among the systems; for example, F10G10 and F10G7S3 exhibit higher *K*_3_ values than LHPC, while F20 shows the lowest. These results suggest that some blended systems possess enhanced long-term reaction capacity in the diffusion-controlled stage, although the extent of this effect depends on blend composition.

### 4.2. Scheffé Mixture-Regression Model and Ternary Response of Mechanical Properties

With the LHPC fraction fixed at 80 wt.% and the total supplementary cementitious materials (SCMs) fixed at 20 wt.%, a first-order Scheffé mixture-regression model was established for the FA–GGBS–SF ternary system to describe the global compositional trend of 28-day compressive strength within the investigated mixture domain and to support preliminary mixture optimization. The model was obtained by regression fitting and is intended for interpolation within the experimental domain only; therefore, it should not be extrapolated beyond the investigated composition range.

Under the mixture constraint *x*_1_ + *x*_2_ + *x*_3_ = 1, the predicted 28-day compressive strength *Y* is given by Equation (5), where *Y* is in MPa and *x*_1_, *x*_2_ and *x*_3_ are the normalized mass fractions of FA, GGBS and SF within the fixed 20% SCM portion:

(5) $$Y=44.7127x_{1}+57.1527x_{2}+65.6112x_{3},$$

where *Y* is the predicted 28-day compressive strength (MPa) and *x*_1_, *x*_2_, and *x*_3_ are the normalized mass fractions of FA, GGBS, and SF, respectively, within the fixed 20 wt.% SCM portion. The coefficient of determination of the model is *R*^2^ = 0.691, indicating that Equation (5) is suitable for representing the overall strength variation trend in the FA–GGBS–SF ternary system under the present fixed-LHPC and fixed-SCM conditions.

**Figure 10:**
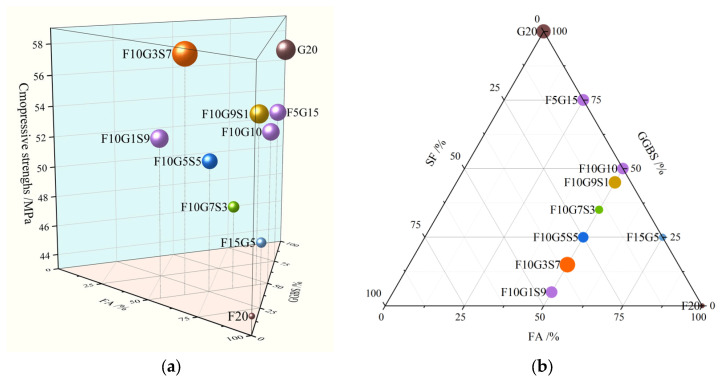
28 d compressive strength landscape in the FA–GGBS–SF ternary compositional space: (**a**) 3D view; (**b**) 2D projection.

Figure 10 illustrates the distribution of 28 d compressive strength within the FA–GGBS–SF ternary space. The minimum is located toward the FA-rich end, while C28 increases as the composition shifts toward the GGBS-rich end, indicating that increasing GGBS elevates the baseline 28-day strength, whereas higher FA tends to depress the lower bound. When FA is fixed at 50% of the SCM portion, the combined use of GGBS and SF produces a non-monotonic response: a local maximum occurs near the GGBS–SF co-blending region, which indicates an effective dosage window for SF under the present 20% SCM framework.

### 4.3. Association Between Hydration Heat Evolution and Strength Development

**Figure 11:**
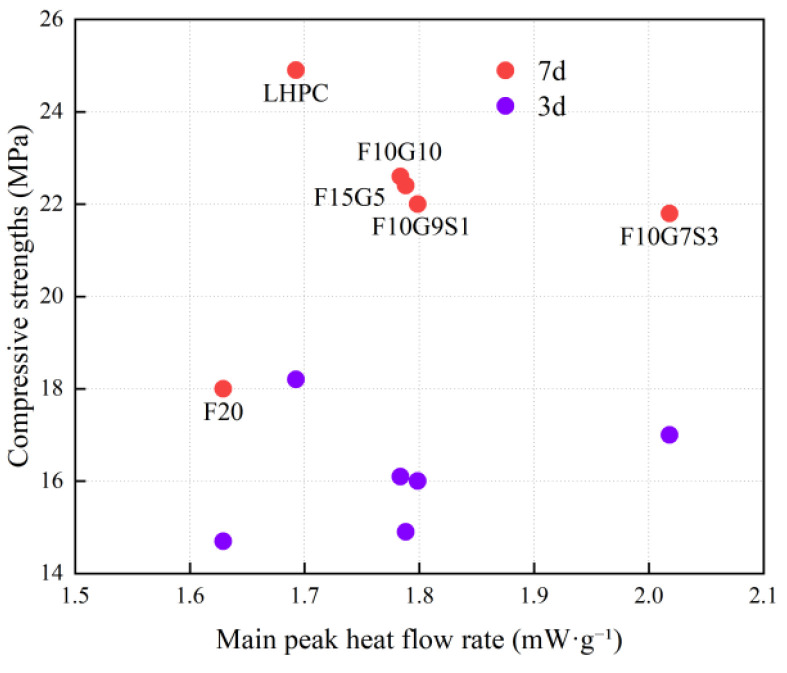
Relationship between main peak heat flow rate and compressive strength at different ages.

**Figure 12:**
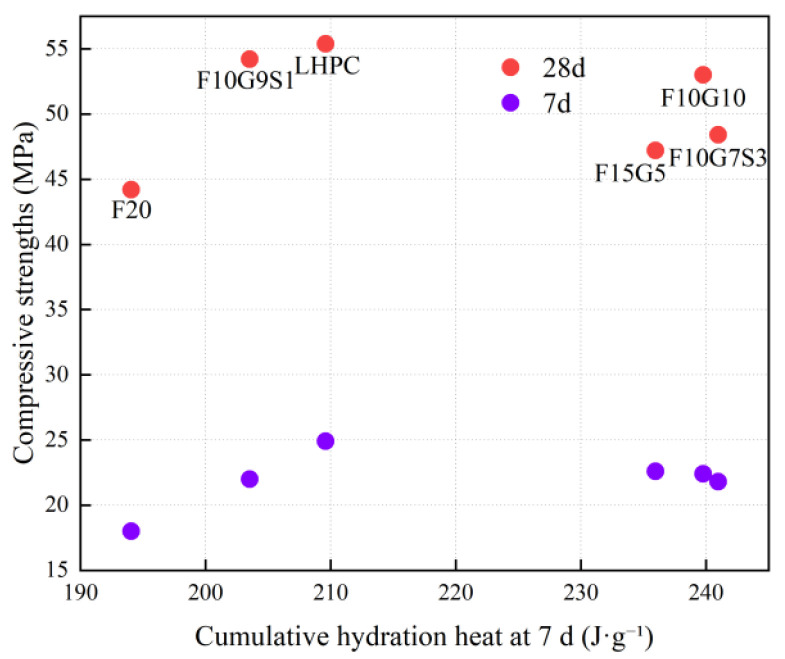
Relationship between cumulative hydration heat at 7 d and compressive strength.

To clarify the link between heat evolution and strength development, the main heat-flow peak rate and the 7-day cumulative heat release obtained from isothermal calorimetry were correlated with compressive strengths at different ages (Figure 11 and Figure 12). Figure 11 indicates that the 3 d compressive strength within the tested range of 14.7–18.2 MPa shows no clear sensitivity to the main heat-flow peak rate. The 7 d strength increases overall as the peak rate rises from 1.63 to approximately 1.78–1.80 mW·g^−1^, as observed from F20 to the blended systems, whereas a further increase to 2.018 mW·g^−1^ for F10G7S3 does not lead to a corresponding strength gain. These results suggest that the main heat-flow peak rate primarily reflects early reaction intensity during the dominant hydration stage, but it is insufficient to determine the macroscopic strength level, particularly at later ages.

Figure 12 further shows a clearly non-monotonic relationship between the 7-day cumulative hydration heat and compressive strength. F20 exhibits both a low 7-day cumulative heat release of 194.06 J·g^−1^ and the lowest 28 d strength of 44.2 MPa, indicating an insufficient overall reaction extent in the fly-ash-only system. For blended systems, when the 7-day cumulative hydration heat varies within approximately 203–241 J·g^−1^, the 28 d strength does not increase linearly with heat release. F15G5 and F10G7S3 show relatively high heat output but only 47.2 and 48.4 MPa at 28 d, respectively, whereas F10G9S1 achieves a high 28 d strength of 54.2 MPa despite a lower cumulative heat release of 203.545 J·g^−1^. This behavior indicates a decoupling between heat release and strength development and highlights differences in heat-to-strength conversion efficiency among mixtures.

**Figure 13:**
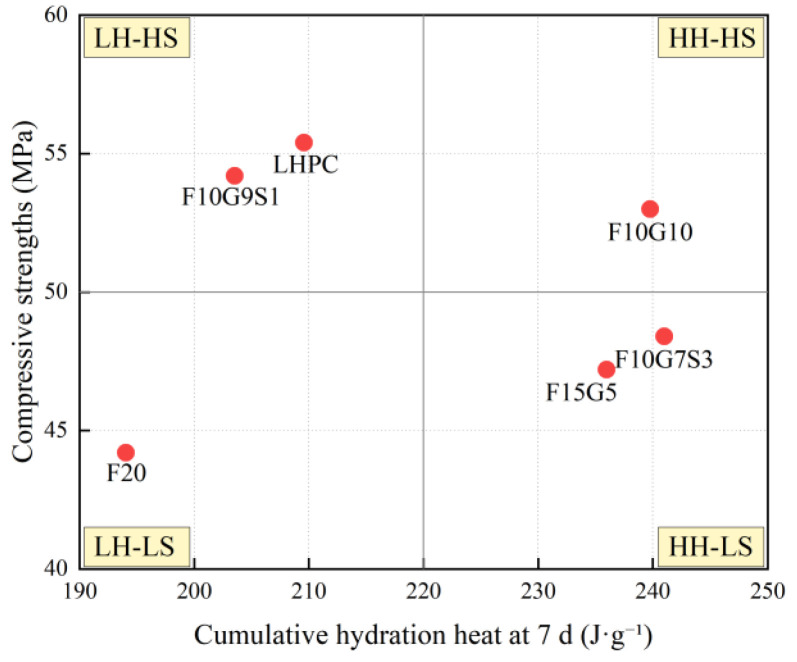
Quadrant classification of mixtures based on cumulative hydration heat and compressive strength.

To further visualize this decoupling, Figure 13 classifies the mixtures into four quadrants using threshold values of 220 J·g^−1^ for the 7-day cumulative hydration heat and 50 MPa for the 28 d compressive strength, corresponding to low heat–high strength, low heat–low strength, high heat–high strength, and high heat–low strength regions. In this framework, F10G9S1, together with the reference LHPC, falls into the low heat–high strength quadrant, whereas F15G5 and F10G7S3 are located in the high heat–low strength quadrant. These results demonstrate that strength development depends not only on the amount of heat released but also on the nature and structural contribution of hydration products. Therefore, the heat–strength relationship should be interpreted in conjunction with Section 4.4, which addresses the association between hydration products, microstructural densification, and strength development.

### 4.4. Association Between Hydration Products, Microstructural Densification, and Strength Development

**Figure 14:**
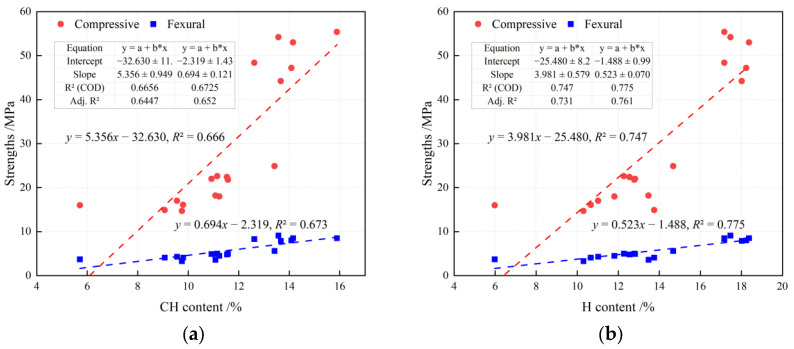
Relationships between hydration products and mechanical strength: (**a**) CH content; (**b**) H content *. * The dashed lines represent the linear regression fitted lines of the corresponding data points.

Figure 14 correlates the TGA-derived portlandite (CH) and bound-water contents with compressive and flexural strengths. CH shows only moderate correlations (R^2^ ≈ 0.66–0.67), consistent with CH being a hydration-progress indicator that is sensitive to dilution and later pozzolanic consumption. In contrast, bound water correlates more strongly with both strengths (R^2^ ≈ 0.75–0.78), highlighting that the “low-heat yet high-strength” pathway is governed by a higher efficiency in converting heat evolution into effective hydrate formation and microstructural densification.

Based on the 3 d XRD patterns in Figure 5a (Section 3.3.1), CH and AFt are identified together with pronounced reflections of unhydrated C3S and C2S, confirming incomplete clinker hydration at this age. Despite the same curing time, the phase assemblage already aligns with divergent performance pathways: F20 represents an under-reacted low-heat/low-strength boundary, F10G9S1 exemplifies a high-efficiency low-heat/high-strength route, whereas F15G5 illustrates a low-efficiency case in which higher heat release does not translate into an effective load-bearing structure. These phase-level observations support the TGA-based inference above and motivate the subsequent SEM evidence of mixture-dependent densification pathways.

**Figure 15:**
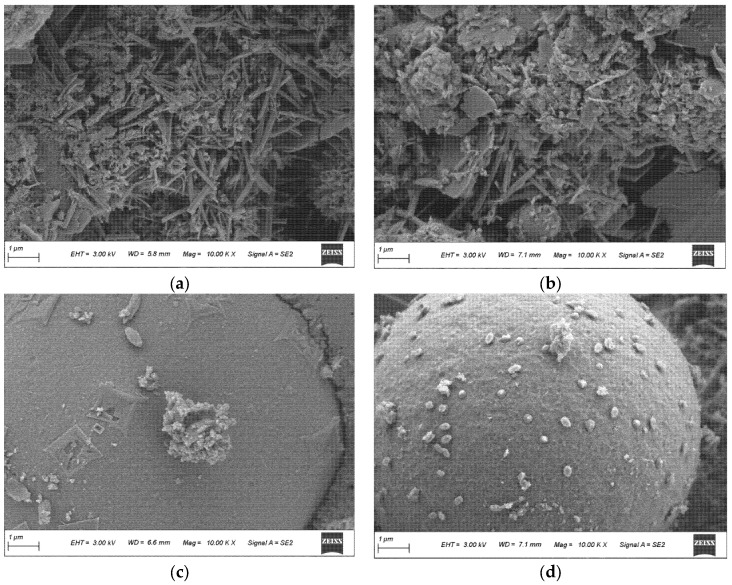
SEM micrographs of representative binders at 3 d and 28 d: (**a**) F15G5-3d; (**b**) F10G9S1-3d; (**c**) F15G5-28d; (**d**) F10G9S1-28d.

The SEM comparison in Figure 15a–d indicates distinct densification pathways for F15G5 and F10G9S1. At 3 d, F15G5 is dominated by needle-like hydrates with a less continuous matrix and more open voids, whereas F10G9S1 exhibits more gel-like features with evident wrapping/filling, resulting in a more continuous microstructure. By 28 d, deposits on fly ash in F15G5 remain localized and non-uniform, while F10G9S1 shows more extensive and uniformly distributed fine deposits, consistent with more effective fly-ash-involved secondary hydration and pore refinement. These observations agree with the stronger bound-water–strength correlation in Figure 14 (higher R^2^) and the 3 d XRD evidence of CH/AFt coexisting with unhydrated clinker, supporting that the low-heat yet high-strength route is controlled by a higher efficiency in converting heat evolution into effective hydrates and microstructural densification rather than by higher heat output alone.

## 5. Conclusions

1. Replacing 20% of LHPC with fly ash, namely F20, reduces both early-age and later-age mechanical performance and thus defines a low-heat and low-strength boundary for the present design domain. At 28 d, F20 reaches 44.2 MPa in compressive strength and 7.9 MPa in flexural strength, whereas LHPC reaches 55.4 MPa and 8.5 MPa, respectively.
2. Under the same total SCM dosage of 20%, replacing fly ash with GGBS substantially restores strength and elevates the baseline performance of LHPC-based blends. Along the composition sequence from FA-dominant to GGBS-dominant mixtures, namely F20, F15G5, F10G10, F5G15, and G20, the 28 d compressive strength increases from 44.2 MPa to 56.7 MPa, indicating that increasing GGBS effectively raises the strength floor.
3. Incorporating silica fume produces a non-monotonic strength response and reveals an effective dosage window. The highest 28 d compressive strength is obtained for F10G3S7 at 58.2 MPa, whereas an excessive silica fume dosage, represented by F10G1S9, leads to a strength reduction, demonstrating that silica fume is beneficial only within an optimized range rather than providing a proportional increase.
4. Isothermal calorimetry shows that multi-component blending modifies early hydration kinetics and heat-release characteristics. Compared with LHPC, which reaches the main hydration peak at 13.505 h, blended mixtures exhibit an earlier peak occurrence in the range of 11.905–12.989 h and a higher peak heat flow, reaching 2.018 mW·g^−1^ for F10G7S3. The 7 d cumulative heat spans 203.545–240.985 J·g^−1^, and F10G9S1 achieves a relatively low 7 d cumulative heat of 203.545 J·g^−1^ while maintaining a 28 d compressive strength of 54.2 MPa, representing a high-efficiency pathway that combines low hydration heat with high strength.
5. Krstulović–Dabić kinetic analysis confirms that the blends differ not only in ultimate heat potential but also in the progression rate of hydration. Fly ash alone reduces the ultimate heat potential to a *Q*_max_ of 217.98 J·g^−1^, which is 7.7% lower than that of LHPC, whereas blends containing GGBS and silica fume increase *Q*_max_ to 269–272 J·g^−1^. In addition, *t*_50_ is extended to about 32 h for F15G5 and F10G10 but shortened to 24.84 h for F10G9S1, indicating mixture-dependent acceleration and retardation behaviors beyond simple dilution.
6. Micro-chemical quantification and microstructural observations indicate that strength development is governed primarily by densification efficiency rather than portlandite accumulation. At 28 d, the bound-water index normalized to LHPC, *I*_H_, reaches 21.46–22.97% in blended systems, higher than 17.17% for LHPC, and bound water shows stronger correlations with strength, with R^2^ in the range of 0.75–0.78, than CH, with *R*^2^ in the range of 0.66–0.67. SEM further confirms a more continuous gel-rich matrix and more uniform deposits in the high-efficiency mixture F10G9S1, supporting that the low-heat and high-strength pathway is controlled by effective hydrate formation and pore refinement rather than by a higher heat output alone.

Overall, within the present fixed-design domain (80 wt.% LHPC and 20 wt.% total SCMs), the low-heat/high-strength performance of LHPC-based blended binders is governed not by heat release alone but by the coupled effects of hydration efficiency, effective hydrate formation, and matrix densification. The results indicate that an appropriate balance among FA, GGBS, and SF can create a favorable compositional window in which reduced hydration heat is achieved without a proportional loss of mechanical performance. In particular, the combined calorimetric, kinetic, TGA, and SEM evidence demonstrates that later-age strength gain depends more strongly on efficient heat-to-hydrate conversion and microstructural refinement than on a higher total heat output. These findings provide both mechanistic insight and practical guidance for proportioning LHPC-based binders for concrete applications requiring early-age temperature-crack control.

It should also be noted that the optimized compositional window identified in this study is specific to the present LHPC–FA–GGBS–SF binder system, raw-material sources, and testing conditions and, therefore, should not be directly generalized to other concrete systems without further verification. Future work should focus on validating the proposed low-heat/high-strength pathway under different raw-material sources, water-to-binder ratios, and practical concrete conditions as well as assessing its durability and engineering applicability at larger scales.

## Figures and Tables

**Table 1:** Chemical composition of raw materials (wt.%).

Oxide	LHPC	FA	GGBS	SF
CaO	59.2	2.46	34.82	0.47
SiO_2_	23.3	44.54	32.93	95.99
Al_2_O_3_	4.86	45.96	19.24	0.99
Fe_2_O_3_	5.59	2.91	0.64	0.2
MgO	2.12	0.46	8.08	0.92
SO_3_	3.35	0.76	2.38	0.16
R_2_O *	0.66	0.63	0.83	0.73
Others	0.92	2.28	1.08	0.54

* R_2_O represents the alkali equivalent, calculated as Na_2_O + 0.658 K_2_O, where K_2_O is converted into Na_2_O equivalent based on molecular weight.

**Table 2:** Elemental composition of raw materials determined by XRF (wt.%).

Element	LHPC	FA	GGBS	SF
Ca	69.09	4.49	48.49	1.09
Si	14.63	43.13	24.27	93.53
Al	3.38	40.77	14.99	0.97
Fe	7.39	5.73	1.05	0.46
Mg	1.66	0.44	6.86	0.97
Na	0.15	0.15	0.46	0.36
K	0.63	1.04	0.58	1.94
S	1.91	0.74	1.65	0.2
Cl	0.04	–	0.09	0.13
Others *	1.12	3.51	1.56	0.35

* Others include Ni, Cu, Zn, Ga, Rb, Sr, Zr, Mo and Pb.

**Table 3:** Mixture nomenclature and binder compositions of the investigated systems (wt.%).

Mixture ID	LHPC	FA	GGBS	SF
LHPC	100	0	0	0
F20	80	20	0	0
F15G5	80	15	5	0
F10G10	80	10	10	0
F5G15	80	5	15	0
G20	80	0	20	0
F10G9S1	80	10	9	1
F10G7S3	80	10	7	3
F10G5S5	80	10	5	5
F10G3S7	80	10	3	7
F10G1S9	80	10	1	9

**Table 4:** Key isothermal calorimetry parameters of LHPC-based binders.

Samples	Time to Main Peak (h)	Main Peak Heat Flow (mW·g^−1^)	Cumulative Heat (J·g^−1^)	
			3 d	7 d
LHPC	13.505	1.6925	166.955	209.595
F20	13.5222	1.62915	158.205	194.06
F15G5	12.9886	1.78365	178.185	235.975
F10G10	12.9528	1.78815	179.26	239.77
F10G9S1	12.4417	1.7987	162.775	203.545
F10G7S3	11.905	2.0183	181.135	240.985

**Table 5:** LHPC-normalized calcium hydroxide index (*I*_CH_) and bound-water index (*I*_H_) of different samples.

Samples	*I*_CH_ (%)			*I*_H_ (%)		
	3 d	7 d	28 d	3 d	7 d	28 d
LHPC	11.1	13.4	15.9	13.5	14.7	17.2
F20	12.2	14.0	17.1	12.9	14.8	22.5
F15G5	12.2	13.9	17.6	13.3	15.3	22.8
F10G10	11.3	14.4	17.7	17.2 *	15.7	23.0
F10G9S1	7.1	13.7	17.0	7.5	16.0	21.8
F10G7S3	11.9	14.5	15.8	13.8	16.0	21.5

* The 3 d *I*_H_ value of F10G10 should be interpreted with caution because the low-temperature mass-loss anomaly below 100 °C may be affected by residual ethanol or ethanol–water volatilization.

**Table 6:** Mean kinetic parameters obtained from K–D model fitting of the hydration process for different binder systems *.

Samples	*Q*_max_ (J/g)	*t*_0_ (h)	*t*_50_ (h)	*n*	*K*_1_	*K*_2_	*K*_3_
LHPC	236.19	2.1606	27.21	1.568524	0.035006	0.009172	0.001782
F20	217.98	2.0389	25.08	1.595054	0.036221	0.009716	0.001643
F15G5	268.77	1.9942	32.81	1.562598	0.033234	0.008165	0.001957
F10G10	270.7	2.0917	32.36	1.494044	0.032873	0.008162	0.00213
F10G9S1	225.54	2.175	24.84	1.555048	0.038939	0.009908	0.001969
F10G7S3	271.55	2.2161	31.77	1.57905	0.037258	0.008453	0.002122

* The reported values are fitted kinetic parameters obtained from the average calorimetric results of three independent tests for each mixture.

## Data Availability

The original contributions presented in the study are included in the article, further inquiries can be directed to the corresponding authors.

## Abbreviations

The following abbreviations are used in this manuscript:

LHPC	Low-heat Portland cement
FA	Fly ash
GGBS	Ground granulated blast-furnace slag
SF	Silica fume
SCMs	Supplementary cementitious materials
w/b	Water-to-binder ratio
XRF	X-ray fluorescence spectroscopy
XRD	X-ray diffraction
TGA	Thermogravimetric analysis
TG	Thermogravimetry
DTG	Derivative thermogravimetry
SEM	Scanning electron microscopy
CH	Calcium hydroxide (portlandite)
AFt	Ettringite
C-(A)-S-H	Calcium (alumino-)silicate hydrate
C_3_S	Tricalcium silicate
C_2_S	Dicalcium silicate
K–D	Krstulović–Dabić model

## Appendix A. Statistical Summary of Flexural and Compressive Strengths

This appendix provides the statistical summary of the mechanical-property results discussed in Section 3.1. The reported values include the mean strength, standard deviation (SD), and coefficient of variation (COV) at different curing ages. These tables are provided to supplement the discussion in the main text and to improve the transparency of the strength-data presentation.

**Table A1:** Mean flexural strength, standard deviation (SD), and coefficient of variation (COV) of the investigated mixtures at different curing ages.

Samples	3 d Mean	3 d SD	3 d COV (%)	7 d Mean	7 d SD	7 d COV (%)	28 d Mean	28 d SD	28 d COV (%)
LHPC	4.7	0.2	4.4	5.9	0.4	5.9	8.6	0.8	9.6
F20	3.3	0.1	4.3	4.5	0.0	0.0	7.9	0.1	1.8
F15G5	4.4	0.7	16.1	5.1	0.2	4.2	8.0	0.1	0.9
F10G10	4.1	0.4	8.7	4.9	0.2	4.4	8.5	0.3	3.3
F5G15	4.2	0.4	10.1	5.4	0.6	11.9	8.9	0.1	0.8
G20	3.9	0.1	1.8	4.8	0.0	0.0	8.6	0.0	0.0
F10G9S1	3.7	0.3	7.6	4.9	0.0	0.0	9.1	0.1	1.6
F10G7S3	4.3	0.1	1.7	5.1	0.5	9.8	8.3	0.6	6.8
F10G5S5	4.0	0.1	3.5	4.8	0.2	4.5	8.4	0.7	8.4
F10G3S7	3.6	0.0	0.0	4.9	0.0	0.0	8.8	0.5	5.7
F10G1S9	3.6	0.1	2.0	4.7	0.1	1.5	8.1	0.6	7.0

**Table A2:** Mean compressive strength, standard deviation (SD), and coefficient of variation (COV) of the investigated mixtures at different curing ages.

Samples	3 d Mean	3 d SD	3 d COV (%)	7 d Mean	7 d SD	7 d COV (%)	28 d Mean	28 d SD	28 d COV (%)
LHPC	17.0	1.8	10.4	22.9	1.0	4.3	51.2	1.0	2.0
F20	14.7	0.3	1.8	18.0	0.6	3.1	44.2	3.3	7.6
F15G5	15.4	1.3	8.7	22.6	0.2	0.7	47.2	3.7	7.8
F10G10	14.9	0.4	2.3	22.5	1.0	4.6	53.0	2.6	4.8
F5G15	17.5	1.2	6.6	23.0	1.0	4.2	53.3	4.3	8.0
G20	16.9	1.0	5.7	22.4	1.0	4.4	56.8	1.5	2.7
F10G9S1	16.0	0.4	2.7	22.0	0.6	2.8	54.2	2.1	3.9
F10G7S3	17.0	0.2	1.3	21.8	0.5	2.5	48.4	7.9	16.3
F10G5S5	15.3	0.3	2.1	23.9	0.6	2.4	51.5	3.5	6.9
F10G3S7	14.4	0.7	5.1	18.9	2.1	11.3	58.2	1.3	2.2
F10G1S9	14.2	0.4	2.7	22.6	0.8	3.4	53.2	9.5	17.8
